# Supplementary material for: Efficacy of Xanthine Oxidase Inhibitors in Lowering Serum Uric Acid in Chronic Kidney Disease: A Systematic Review and Meta-Analysis
Source: J Clin Med. 2022 Apr 27;11(9):2468. doi: 10.3390/jcm11092468 (PMC9105680; doi:10.3390/jcm11092468)
Supplement: Supplementary file 1 [file jcm-11-02468-s001.zip › jcm-1659205-supplementary.pdf]

Supplementary Material

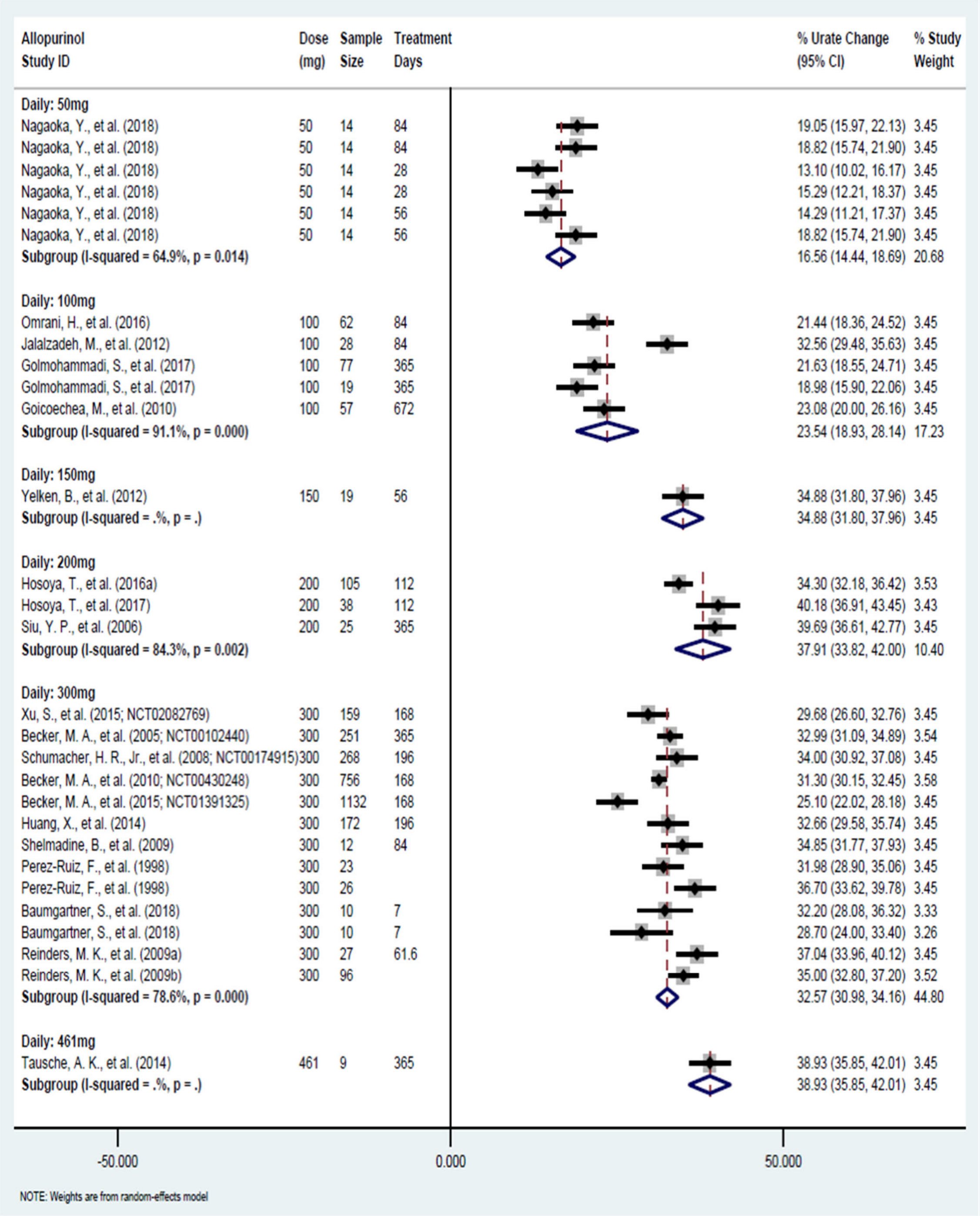

Figure S1. Forest plot of SUA-lowering effect of allopurinol [42,43,45–63].

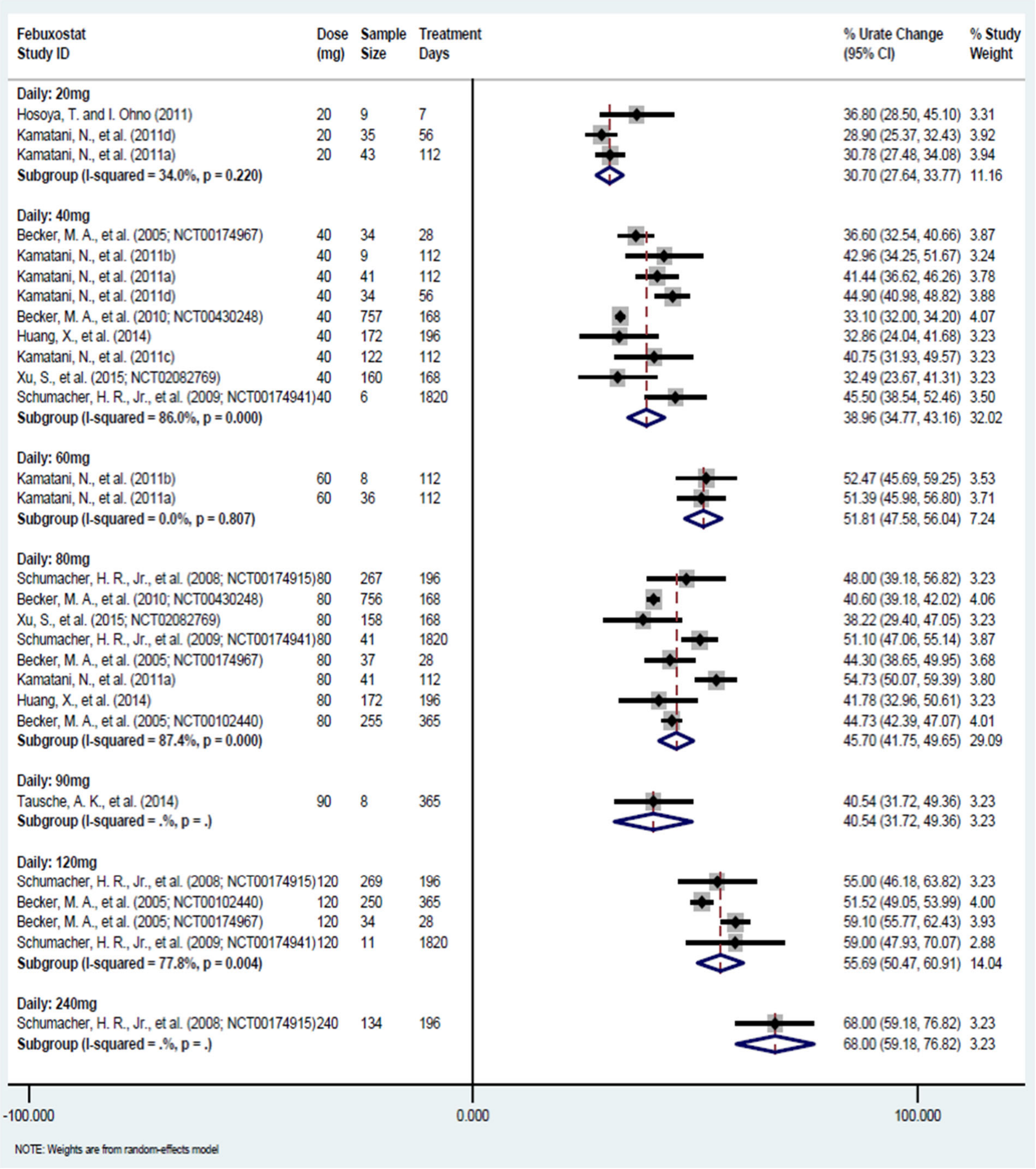

Figure S2. Forest plot of SUA-lowering effect of febuxostat [42,43,45–47,51,64–70].

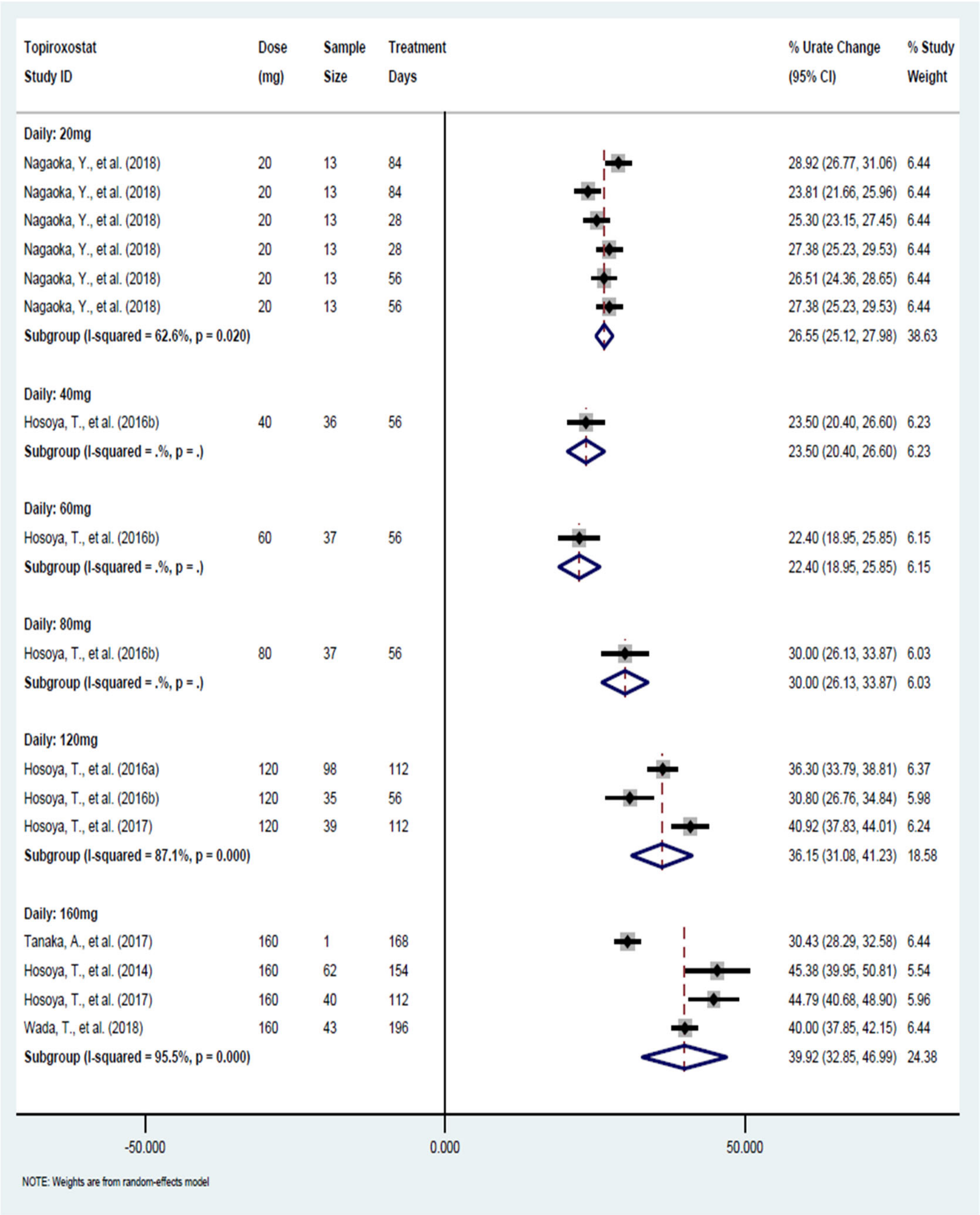

Figure S3. Forest plot of SUA-lowering effect of topiroxostat [49,50,63,71–74].

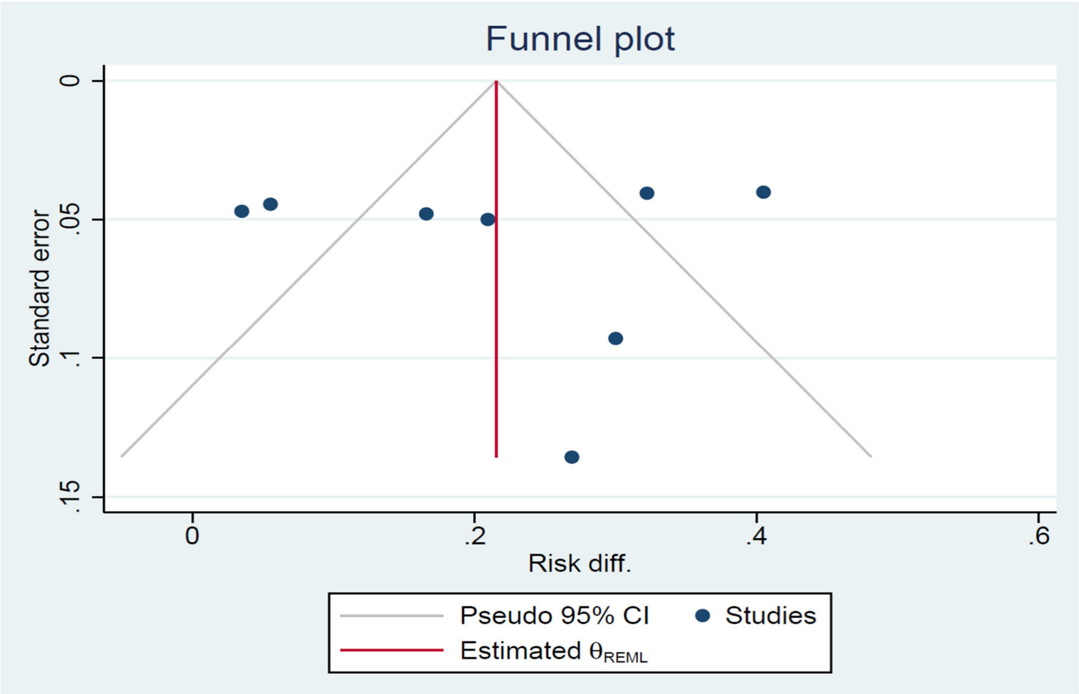

Figure S4. Funnel plot of AD for febuxostat monotherapy vs. allopurinol 300 mg daily [42–45].

Table S1. Exclusion of the Trial Arms in the Urate-Lowering Therapy Drug Analysis.

		Reasons for Exclusion of the Trial Arms						
**Uric Lowering Therapy Drugs**	**Collected Number of Trial Arms**	**Normal Range of Serum Uric Acid**	**Dosage Other than Once Daily (no QD)**	**Dose Titration**	**Uric Lowering Data Availability**	**CKD Diagnosis Availability**	**Placebo or Colchicine Trial Arms**	**Included Number of Trial Arms in the Final Analysis**
Allopurinol	53	5	5	8	5	1		29
Benzbromarone	16		4		3	5		4
Febuxostat	48	6	1	2	11			28
Lesinurad	9	7			1			1
Pegloticase	11	4	7					0
Probenecid	4	1	1			1		1
Rasburicase	22	8	1	1	12			0
Topiroxostat	21	1	4					16
Combination of two ULT drugs	26	9	1	9	3			4
Colchicine	2						2	0
Placebo	23						23	0
